# Hantavirus Pulmonary Syndrome Caused by Maripa Virus in French Guiana, 2008–2016

**DOI:** 10.3201/eid2310.170842

**Published:** 2017-10

**Authors:** Séverine Matheus, Hatem Kallel, Claire Mayence, Laetitia Bremand, Stéphanie Houcke, Dominique Rousset, Vincent Lacoste, Benoit de Thoisy, Didier Hommel, Anne Lavergne

**Affiliations:** Institut Pasteur de la Guyane, Cayenne, French Guiana (S. Matheus, L. Bremand, D. Rousset, V. Lacoste, B. de Thoisy, A. Lavergne);; Centre Hospitalier de Cayenne, Cayenne (H. Kallel, C. Mayence, S. Houcke, D. Hommel)

**Keywords:** hantavirus, Maripa virus, French Guiana, human cases, hantavirus pulmonary syndrome, viruses, lungs

## Abstract

We report 5 human cases of hantavirus pulmonary syndrome found during surveillance in French Guiana in 2008–2016; of the 5 patients, 4 died. This pathogen should continue to be monitored in humans and rodents in effort to reduce the occurrence of these lethal infections in humans stemming from ecosystem disturbances.

New World hantavirus pulmonary syndrome (HPS) is an emerging infectious disease caused by viruses of the family *Bunyaviridae* and genus *Hantavirus* (*1*). Hantaviruses are transmitted to humans most frequently by inhalation of viral particles expired by chronically infected rodents. Since the first case reported in the United States in 1993, HPS has been reported in Argentina, Chile, Brazil, Uruguay, Paraguay, Bolivia, and many other countries in South and Central America (*2**,**3*).

The circulation of hantavirus in French Guiana, a French overseas department located in the Amazon rainforest on the northeastern corner of South America, was first suggested in a retrospective serologic survey that showed an antibody prevalence of 1.42% among a select population of 420 patients having symptoms consistent with HPS (*4*). In 2008, after publication of the antibody prevalence data, active surveillance for this emerging infectious disease in humans was implemented, and in that same year, the first native biologically confirmed human HPS case occurred (*5*). The novel hantavirus detected in that patient was closely related to the Rio Mamoré virus and was named Maripa virus (*6*). We report the results of this 8-year hantavirus surveillance in humans and rodents in French Guiana and an investigation for hantavirus in rodents.

## The Study

During 2008–2016, samples from 151 patients having symptoms consistent with hantavirus disease were received for diagnosis at the Institut Pasteur de la Guyane, Cayenne, French Guiana. Patient symptoms were primarily fever, myalgia, headache, and cough; most patients were hospitalized. We conducted serologic and molecular investigations with patient samples, depending on the time of sample collection; for samples collected days 0–7 after illness onset, we performed both investigations, and for samples collected thereafter, only serology. These analyses led to the identification of 5 patients with acute hantavirus infection that occurred in August 2008, December 2009, December 2010, May 2013, and October 2016 and 3 patients with hantavirus IgG only.

All 5 patients with acute infection were men (Table) who did not have a history of travel outside French Guiana. Each arrived at the emergency department of Andrée Rosemon General Hospital in Cayenne with a rapid onset of acute lung injury requiring admission to the intensive care unit (ICU) for intubation and mechanical ventilation. Of the 5 patients, 3 had a history of other medical conditions: 2 had hypertension and 1 had diabetes. All 5 patients had the initial symptoms of fever and dyspnea, 4 had myalgia, 3 had cough, and 2 had diarrhea and vomiting. At admission to the ICU, all patients had the following clinical characteristics: hemodynamic changes, acute lung and kidney injury, lactic acidosis, elevated hematocrit, standard or low protein level, and thrombocytopenia (Table). Laboratory tests for infectious agents ruled out malaria, dengue, leptospirosis, Chagas disease, Q fever, cytomegalovirus, and HIV, and blood cultures for bacterial growth were all negative. Chest radiographs of all patients showed a bilateral alveolar infiltrate with pleural effusion. Heart sizes were within reference limits. At admission to the ICU, the patients received mechanical ventilation, fluid infusion, and catecholamines. Four of the 5 patients died within the first 24 hours after admission (case-fatality rate 80%). The surviving patient was discharged from the hospital 47 days after admission with complete clinical recovery (*5*).

**Table:** Clinical characteristics of 5 male patients infected with Maripa virus, French Guiana, 2008–2016*

Characteristic	Reference range	Patient					Mean (min–max)	SD	No. (%) patients
		1	2	3	4	5			
Year case reported		2008	2009	2010	2013	2016			
Age, y		38	56	49	67	71	56.2 (38.0–71.0)	13.4	
Location of residence		Tonate-Macouria	Rémire-Montjoly	Tonate-Macouria	Tonate-Macouria	Iracoubo			
Day of hospital admission after symptom onset		7	4	2	4	4	4.2 (2.0–7.0)	1.8	
Characteristics at admission to ICU									
Heart rate, beats/min		140	150	140	168	170	154 (140–170)	15	
Shock		Yes	Yes	Yes	Yes	Yes			5 (100)
Acute lung injury		Yes	Yes	Yes	Yes	Yes			5 (100)
Acute kidney injury		Yes	Yes	Yes	Yes	Yes			5 (100)
Urea nitrogen, mmol/L	1.7–8.3	9.3	10.0	10.7	13.7	6.4	10.0 (6.4–13.7)	2.6	
Creatinine, µmol/L	62–106	192	174	196	196	126	176.8 (126.0–196.0)	29.8	
Serum protein, g/L	60–80	44.8	69.0	30.1	57.0	55.0	51.2 (30.1–69.0)	14.6	
Lactate, mmol/L	0.63–2.44	2.2	8.0	5.3	3.1	5.0	4.7 (2.2–8.0)	2.2	
Leukocytes, ×10^9^ cells/L	4–10	22.5	21.1	19.5	9.6	9.7	16.5 (9.6–22.5)	6.3	
Platelet count, ×10^9^/L	150–400	50	149	67	125	79	94.0 (50.0–149.0)	41.5	
Hematocrit, %	38–51	50.5	66.6	55.9	52.6	41.7	53.5 (41.7–66.6)	9.0	
AST, IU/L	<37	17	49	10	18	17	22.2 (10.0–49.0)	15.3	
ALT, IU/L	<40	31	47	24	58	29	37.8 (24.0–58.0)	14.2	
Bilirubin, µmol/L	<17	3.4	8.2	6.0	6.0	4.6	5.6 (3.4–8.2)	1.8	
CPK, U/L	38–174	215	119	149	181	262	185.2 (119.0–262.0)	55.9	
Troponin, µg/L	<0.1	ND	ND	0.20	0.06	0.25	0.17 (0.06–0.25)	0.10	
CRP, mg/L	<10	192.0	166.0	93.3	92.4	154	139.5 (92.4–192.0)	44.8	
Death		No	Yes	Yes	Yes	Yes			4 (80)
Time of death after admission, h		NA	2	6	16	14	9.5 (2.0–16.0)	6.6	

*ALT, alanine aminotransferase; AST, aspartate aminotransferase; CPK, creatine phosphokinase; CRP, C-reactive protein; ICU, intensive care unit; max, maximum; min, minimum; NA, not applicable; ND, not determined.

We evaluated patient serum samples collected at admission to either the emergency department or the ICU to confirm a suspected hantavirus infection. We performed serologic analyses with an indirect ELISA using inactivated Sin Nombre virus (SNV) antigen designed to detect IgM and an ELISA using recombinant SNV antigen designed to detect IgG, both provided by the US Centers for Disease Control and Prevention (*7*). The results revealed the presence of IgM and IgG reactive against SNV antigens in all samples. We confirmed acute infection by detecting a fragment of the small (S) segment (encoding the nucleoprotein) by reverse transcription PCR using consensus primers targeting New World hantavirus (*8*). We confirmed the Maripa virus sequence by sequencing the PCR products. Comparison of the partial sequences (393 bps excluding primers) obtained from the last 4 cases confirmed the closeness of these sequences with that obtained from the first case of Maripa virus identified in 2008 (*5*). Finally, we generated the complete RNA sequence of the S segment for the 5 cases and compared it with a panel of New World hantavirus sequences. The 5 sequences of Maripa virus exhibited 96%–100% nucleotide identity among themselves; the largest divergence observed (96%) was between the virus sequences from patients 2 and 5. These sequences also showed 83.6%–85.8% nucleotide identity with other sequences belonging to the Rio Mamoré clade. Phylogenetic relationships were inferred from alignment with 1,308 nt of the S segment by using a Bayesian approach performed with Mr. Bayes 3.2.2 (*9*). All Maripa virus sequences identified in French Guiana clustered together within the Rio Mamoré and Anajatuba clades at the basal position of this group (Figure).

**Figure:**
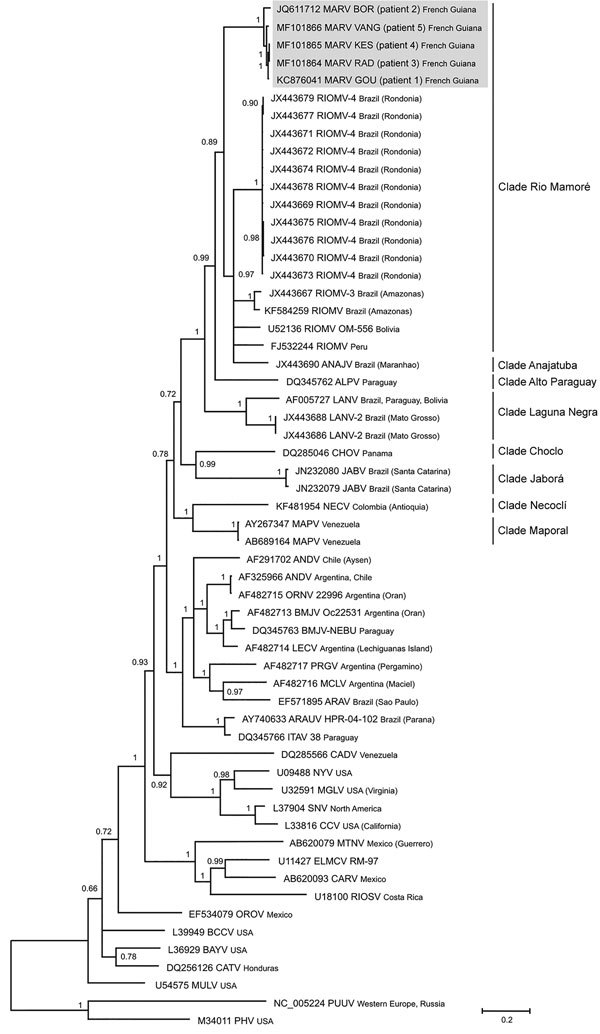
Phylogenetic tree based on the 1,308-bp fragment of the small (S) segment of 58 hantaviruses, including the 5 Maripa hantavirus isolates identified in French Guiana, 2008–2016 (gray shading). Tree was constructed by using the general time-reversible plus gamma distribution plus invariable site model of nucleotide evolution. GenBank accession numbers of viruses are indicated. Support for nodes was provided by the posterior probabilities of the corresponding clades. All resolved nodes have posterior probability >0.7. Scale bar indicates mean number of nucleotide substitutions per site.

During the same period, we also found 3 patients (2 men and 1 woman) positive for hantavirus IgG and negative for virus genomic material by reverse transcription PCR. These serologic results reflected a previously cleared hantavirus infection, given the short time between the appearance of symptoms and sample collection. No data concerning history of potentially severe disease or travel to another country were available for these patients, and infection by another New World hantavirus circulating in the region causing no or mild clinical symptoms could not be ruled out.

We performed environmental investigations around the residences of the 5 patients with confirmed hantavirus infections. Patients 1, 3, and 4 were from different parts of the same municipality, Tonate-Macouria (Table), and potentially exposed to hantavirus at a forest edge (patient 1), a periurban area (patient 3), and a slash-and-burn agricultural field (patient 4). Patient 2 was from Rémire-Montjoly municipality, a suburb of Cayenne (the largest city in French Guiana), and patient 5 was from a slash-and-burn agriculture field in the Iracoubo municipality (Table). No secondary human cases were reported near these confirmed cases. We set up a large ecoepidemiologic survey in French Guiana and captured rodents around the patients’ homes to characterize rodent reservoirs (*10*). This survey led to the capture of 20 rodents of 5 different species. By using molecular approaches, we detected 4 Maripa virus–positive rodents: 2 *Oligoryzomys delicatus* (formerly named *O.* *fulvescens* in French Guiana) (*11*), 1 of which was found near the home of patient 4, and 2 *Zygodontomys brevicauda*, both found near the home of patient 2.

## Conclusions

The results of a retrospective serologic study in French Guiana prompted us to pursue an active human surveillance program focused on patients with clinical symptoms consistent with hantavirus disease. This surveillance, conducted over 8 years, showed that human infection with Maripa hantavirus in this department is rare but associated with a high case-fatality rate. In the context of environmental perturbations with growing and unplanned urbanization and human populations that increasingly come in contact with wild mammalian fauna, surveillance in humans and investigations with rodent reservoirs should continue. These initiatives could help prevent the potential emergence of HPS in French Guiana.
